# The Moral Injury of Ineffective Police Leadership: A Perspective

**DOI:** 10.3389/fpsyg.2022.766237

**Published:** 2022-04-15

**Authors:** Bobbi Simmons-Beauchamp, Hillary Sharpe

**Affiliations:** Graduate Centre for Applied Psychology, Athabasca University, Athabasca, AB, Canada

**Keywords:** police leadership, ineffective police leadership, police culture, police, police officer, moral injury, police trauma, police organisation

## Abstract

Research suggests that Canadian police officers are exposed to trauma at a greater frequency than the general population. This, combined with other operational stressors, such as risk of physical injury, high consequence of error, and strained resources, can leave officers less resilient to organizational stressors. In my experience, a significant and impactful organizational stressor is ineffective leadership, which include leaders who are non-supportive, inconsistent, egocentric, and morally ambiguous. Ineffective leadership in the context of paramilitary police culture has been recognized as psychologically distressing. Further, moral injury may result when leadership fails to meet officers’ needs, expectations, and values. Ineffective leadership and resulting moral injuries are an understudied area in the literature. This review will help provide a comprehensive context of policing and the impact of ineffective leadership on police mental health.

## The Moral Injury of Ineffective Leadership: A Perspective

Policing is a challenging and demanding profession, complicated by societal demands and expectations, the uniqueness of the work environment, and the elevated level of exposure to stress and trauma. In my examination of available literature and research, I learned the overall impact of these factors has been a topic of discussion, however, the study of more specific stressors, such as ineffective leadership, is nascent. I opine that it is important to acknowledge ineffective leadership as a significant stressor that may contribute to police officer moral injury. Moral injury is a response to excessive moral pain resulting in emotional and psychological suffering (Litz and Kerig, 2019; Roth et al., 2022). If left unrecognized, the injury can result in a decline of overall mental health due to the resulting stress, anxiety, and potentially depressive symptoms (Schafer, 2010; Litz and Kerig, 2019; Wolter et al., 2019; Chan and Andersen, 2020; Demou et al., 2020; Roth et al., 2022). Moreover, ineffective leadership, which I argue is morally injurious, can lead to declining mental health and potentially mental illness. In their review, Violanti et al. (2017) demonstrated that police officers may become less resilient due to their disproportionate exposure to operational trauma and stress, so addressing factors within police organizations that can contribute to further psychological suffering is vital.

Organizational stressors, such as limited resources, interpersonal conflict, discrimination, and, significantly, ineffective leadership impact the mental health of police officers, much like operational stressors, which include traumatic incidents, risk of physical injury, and high consequence of error (Schafer, 2010; Wolter et al., 2019; Chan and Andersen, 2020; Demou et al., 2020). Trauma and stress can erode resiliency (Violanti et al., 2014; Andersen et al., 2015; Tabinia and Radecki, 2018; Carlson-Johnson et al., 2020), and when an officer is worn down, coping in a paramilitary police culture (hierarchal, strong discipline, and directed) becomes exceedingly difficult. Once the workforce, and ultimately the organizational police culture, is negatively affected by ineffective leadership, an officer’s ability to cope becomes further compromised. This can lead to a *psychological injury* which is a psychiatric or psychological condition brought on by traumatic exposures and/or stress (Young et al., 2020).

In this perspective I identify and compare operational and organizational stressors with the intent of demonstrating through the literature how organizational stressors, specifically ineffective leadership, injures police officers. As a police officer, I share my experience of how the drain of police work can leave officers unable to endure in a poorly led command environment. I explain how police culture can create a sense of helplessness for officers and can sustain ineffective leadership. I explore the direct effect this type of leadership has in creating moral injury, and how the specific issue warrants further attention. Recognizing the correlation between ineffective leadership and moral injury allows police leadership and clinicians to be better-informed about this aspect of the officer experience. Society relies on police officers to be in optimal mental health because of the critical decisions and judgments they are required to make in the moment. Understanding the context of police work and police culture is necessary when determining how best to approach their psychological needs.

## Operational Stressors vs. Organizational Stressors in Policing

Canadian researchers McCreary and Thompson (2006) developed scaled measurements of police operational stressors and police organizational stressors (McCreary and Thompson, 2006). Qualitative interviews were conducted with members of the Ontario Provincial Police and the resulting themes informed two 20-item scaled questionnaires, respectively, titled Operational Police Stress Questionnaire (PSQ-Op) and the Organizational Police Stress Questionnaire (PSQ-Org; McCreary and Thompson, 2006). Examples of operational stressors measured are shift work, managing social life outside of work, negative comments from the public, and traumatic events (McCreary and Thompson, 2006, p. 617). Organizational stressors measured include dealing with supervisors, internal investigations, inadequate equipment, and bureaucratic red tape. Existing literature demonstrates the importance of recognizing organizational stressors as potentially detrimental to police officer mental health (Schafer, 2010; McCreary et al., 2017; Wolter et al., 2019; Chan and Andersen, 2020; Demou et al., 2020). Chan and Andersen (2020) suggest that organizational stressors can increase risk to mental health and interfere with recovery for those who have experienced psychological injuries.

### Police Operational Stressors

Canadian public safety personnel (PSP) are defined by the Government of Canada as firefighters, paramedics, correctional officers, emergency dispatchers, and police officers (Oliphant, 2016). PSP experience traumatic events more often than the general population. Carleton et al. (2018b) report that 96.6% of PSP have experienced a traumatic or critical event six or fewer times, while 89% have experienced up to 11 traumatic or critical events. By contrast, 50–90% of the general population have lived through one or more traumas in their lifespan (Carleton et al., 2018b). Increased and cumulative exposure to trauma for PSP creates a greater risk for the development of psychological distress or injury. This is a highly studied area and examples of such include Carleton et al. (2018a), Marchand et al. (2015), Papazoglou (2012), and Ricciardelli et al. (2018). The distress and/or traumatization can result in substance abuse (self-medicating), hypervigilance, low mood, anxiety, and other symptoms consistent with mental health issues (Ricciardelli et al., 2018; Carleton et al., 2018a). Psychological distress can also manifest physically through disrupted sleep, nightmares, physical and mental fatigue, headaches, and changes in appetite (Ricciardelli et al., 2018). The ramifications of these symptoms can have significant consequences. Excessive and unmanaged operational stress can lead to poor decision-making, service complaints, risk to police and public safety, and an unhealthy, compromised workforce (Andersen et al., 2015).

### Police Organizational Stressors

McCreary and Thompson (2006) identified 20 organizational PSQ items that include dealing with co-workers, the perception of having to volunteer free time, and being looked down upon when injured. The highest rated items identified in the study are leaders over-emphasizing the negatives (2.71 out of 5), favoritism (3.35 out of 5), inconsistent leadership style (3.38 out of 5), and always having to prove yourself (3.47 out of 5; McCreary and Thompson, 2006, p. 507). These values have been consistent in research conducted by Carleton et al. (2020), Chan and Andersen (2020), and McCreary et al. (2017). Some authors also identify organizational stressors to include limited resources, interpersonal conflict, and, significant to this perspective, ineffective leadership (Schafer, 2010; Demou et al., 2020). Carleton et al.’s (2020) findings highlight the importance of recognizing how “non-traumatic workplace stressors” (p. 19), including both organizational and operational stressors, impact an officer’s mental health. In fact, the authors suggest that these stressors may be more impactful than previously considered. From my own experience, I can state that when these stressors are ignored, exacerbated, or prolonged at the hands of ineffective leaders they can be significantly injurious, more so than any trauma I have been exposed to in my career.

## Moral Injury

Research and empirical evidence are scarce on the topic of moral injury in policing (Papazoglou et al., 2020b). Dedicated attention has mainly been given to military-related contexts (Griffin et al., 2019). Moral injury is the negative impact of moral and ethical challenges faced in the line of duty (Papazoglou and McQuerrey Tuttle, 2018; Litz and Kerig, 2019; Roth et al., 2022). In the world of policing, most often a moral injury is attributed to what an officer has done (officer involved shootings) or what they think they could have done (prevented a death). The experience of moral injury can be overwhelming, affecting how a person thinks, feels, and acts (Griffin et al., 2019). It can result in emotions, such as shame, betrayal, sadness, and anger (Griffin et al., 2019; Litz and Kerig, 2019; Papazoglou et al., 2020a; Roth et al., 2022). Moral injury, if not addressed, can result in other mental health issues or disorders (McEwen et al., 2020). A moral injury is dependent upon the degree of “psychological, social, and spiritual harm and impairment” (Litz and Kerig, 2019, p. 345) along with the frequency of exposure to events that challenge the morality of officers (Litz and Kerig, 2019).

Griffin et al. (2019) suggest moral injury can be evaluated through “the extent to which individuals appraise themselves as victims of another’s transgressive behavior” (p. 355). More specifically, the experience of working under ineffective leadership itself is a *potentially morally injurious event* (PMIE; McEwen et al., 2020) which can result in a moral injury. Roth et al. (2022) state the betrayal type PMIE, in some cases, can be attributed to breach of trust and a leader’s actions being non-congruent with an officer’s values. Papazoglou et al. (2020b) argued that this type of failure in leadership can be the most morally assaultive, which highlights the significant impact ineffective leadership can have on the mental health of officers.

## Police Culture

To fully understand the impact of ineffective police leadership, an understanding of police culture is required. Police culture in Canada has its own identity based on shared values, norms, language, and perspectives grounded in a paramilitary environment. Colonialism provided the foundation of policing based on Eurocentric, male-dominated discourse, and ideals (Drummond-Smith, 2018; Papazoglou and McQuerrey Tuttle, 2018). There is a self-established barrier that separates officers from the rest of society, creating an us-versus-them mentality. It is my experience that the prevalent crime fighter approach to policing sustains this divide. Additionally, barriers can exist within police culture that creates ongoing challenges for women and people of diverse ethnicity. A recent report completed on the culture of the Royal Canadian Mounted Police (RCMP) identified obstacles to a healthy work environment within the RCMP, including sexual harassment and gender or sexual orientation-based discrimination (Bastarache, 2020). Further, systemic racism in policing creates significant barriers in recruiting officers of color (Workman-Stark, 2017), and for those who do serve, the expectation is for them to conform to the pre-existing culture based on Eurocentric ideals (Loftus, 2010). A recent study of the Ontario Provincial Police (OPP) found that over 50% of respondents experienced harassment, bullying, and discrimination (Cunningham et al., 2019). The internal divide sustains the Eurocentric, male-dominated perspectives as police organizations are not typically reflective of the communities they serve.

The power in police culture is held by upper-ranking leadership. Police culture can permit ineffectiveness at the very top to dominate because there are few to hold that leader accountable. Workman-Stark (2017) explained, “Through their words and actions, police executives establish norms about risk taking, health and wellness, employee empowerment, dress and deportment, and the actions that are more favorably viewed in terms of promotions, job assignments, and other types of rewards” (p. 27). Leaders demonstrate what is important by that which they give their attention to and the resulting actions that they take (Workman-Stark, 2017). Leaders set the standard for the workings of an organization, so their ineffectiveness permeates the day-to-day operations, systems, and morale of the membership. Police culture also cultivates the unspoken requirement of rank and status being respected, despite how the officer who holds that rank and status leads, behaves, and/or influences (Drummond-Smith, 2018). This required obedience can create significant stress, insult an officer’s values, and be morally injurious (Drummond-Smith, 2018; Litz and Kerig, 2019; Roth et al., 2022).

The process of assimilating into police culture begins in training and continues throughout an officer’s career. Following rules and regulations becomes unequivocally necessary, despite individual opinion. The context of the police experience is further ingrained when training cadets becomes recruits and begin to work with an officer already established in the field. When this process is complete the individual has been indoctrinated into the collective often defined by scepticism, suspicion, and pessimism (Loftus, 2010; Workman-Stark, 2017). During my career, I have shared the sense of distrusting organizational motivations, not having faith in the morality of the public, being cynical, lack of faith in the justice system, and anticipating negative outcomes. Authoritarianism becomes the framework of an officer’s experience (Workman-Stark, 2017, p. 32).

The uniqueness of this culture also creates an environment of perceived weakness when officers express their emotional challenges, which can be alienating (Papazoglou, 2012; Demou et al., 2020). Police officers themselves have described police culture as cynical and rife with toxic masculinity, where the stigma about mental illness adds another burden to those who are already struggling (Demou et al., 2020). The shame and stigma about declining or poor mental health and mental illness can further traumatize an injured officer (Papazoglou, 2012). Ingram et al. (2013) suggested the “universally shared attitudes, values, and norms” (p. 367) attributed to police culture can aid in coping with the challenges of police work because of the common experience. However, this requires officers to feel safe in disclosing their thoughts, feelings, and emotions. In the 2019 study of the Ontario Provincial Police, only 45% of respondents indicated they felt confident that they would be supported through difficulties with their mental health (Cunningham et al., 2019). While this is only one study, it speaks to the oft-cited shared experience of officers not feeling safe in disclosing mental health challenges at work.

### Ineffective Police Leadership

Police leadership has immense influence on police culture. Leaders set the tone for the mission, values, and care for the employees of police organizations. Their actions create the perception of being supportive or non-supportive, which can affect the mental health of police officers (Schafer, 2010). The nature of a chain of command and of authoritarianism, common traits of police culture, can lead to psychological injuries (Litz and Kerig, 2019; Roth et al., 2022), as I have lived. Leaders who lack stress management and emotional regulation skills may make what appear to be illogical decisions, which can create doubt among their followership. Thus, the command rank structure that demands following leadership, despite an officer’s faith (or lack of faith) in that leadership, can produce significant stress (Sarver and Miller, 2013). Chan and Andersen (2020) support this by stating poor leadership and management can be “psychologically hazardous critical incidents” (p. 496), thus calling attention to the great harm that ineffective leadership can have on subordinates.

To better understand ineffective leadership, Schafer (2010) introduced the idea of evaluating leadership through the concepts of what a leader does and what they fail to do (p. 737). Leaders demonstrate ineffectiveness through traits of deficient behaviors including self-centredness, arrogance, closed mindedness, micromanagement, and putting political concerns and allegiances above the safety and welfare of officers (Schafer, 2010, p. 741–742). These traits do not work in the current reality of policing. In addition, Schafer (2010) suggested ineffective leadership is also demonstrated through a poor work ethic, failing to act when it is appropriate to do so, unproductive communication, failure to interact successfully with others, a lack of integrity, or what a leader does not do (p. 742–743). Ineffective leadership contributes to an officers’ perception that they are helpless and creates an oppressive atmosphere of fear and the felt sense of disinterest (Schafer, 2010).

#### Personal Experience With Ineffective Leadership

The literature has explained inconsistent leadership as frequent changes in management, which has been used as a factor in measuring organizational stressors (McCreary et al., 2017; Carleton et al., 2020). I offer that *inconsistency* in the context of leadership can also be understood as erratic, disorganized, and disengaged, and this leads to ineffectiveness. I have experienced inconsistent leadership in the form of an ever-changing strategic direction and fickle decision-making, resulting from not regularly adhering to organizational values and ethics. I have been led by individuals who present different versions of themselves from one day to the next which creates uncertainty, lack of trust, and doubt of authenticity, all of which combined can negatively influence success in achieving organizational goals. This stated, I offer that inconsistency is an aspect of ineffective leadership. The following are examples of what I have observed and experienced as ineffective leadership:

- Rewarding loyalty to a leader’s personal and professional agendas
- Berating and belittling employees in front of lower ranking officers
- Dishonesty
- Mismanagement of resources, causing a critical front-line staffing crisis
- Minimizing or dismissing concerns about the lack of front-line resources and training leaving a workforce feeling unsafe and not supported
- Leadership actions and inactions creating an unhealthy workforce not capable of effectively coping with line of duty deaths
- Addressing critical staffing shortages only after the death of front-line officers
- Leading through personal bias
- Moral ambiguity

Promotion through the ranks to these influential positions is not always supported with adequate training and professional development, which can set leaders up for failure. Although policing agencies have varied promotional processes, in my experience, these processes pay little attention to a candidate’s emotional intelligence, their ability to support others through crisis, or their ability to manage their own behaviors. I believe lack of leadership capabilities can also be attributed to the individual leader’s own experience with critical or traumatic events, or that they may be injured themselves, which further supports the need to focus on the shortcomings of leadership.

#### The Officer Experience

The challenge for police officers is balancing the operational requirements of the job with the organizational factors, such as ineffective police leadership. Organizational factors beyond an officer’s control may seem “oppressive, unnecessary, and inescapable” (Chan and Andersen, 2020, p. 497). There is a plethora of literature and research concerning posttraumatic stress disorder (PTSD) and first responders, including police, but PTSD is not always the issue (Papazoglou and McQuerrey Tuttle, 2018; Ricciardelli et al., 2018; Santa Maria et al., 2018; Carleton et al., 2018b; Chan and Andersen, 2020). Many police officers experience declining mental health, but do not meet the criteria for PTSD (Pietrzak et al., 2012). A lack of personal coping strategies can increase the risk of psychological injuries, including moral injury. The ability to remain resilient in the face of ineffective leadership relies upon an officer’s protective factors and life context (Chan and Andersen, 2020).

## Future Research

What is lacking in existing literature is not only further research concerning the impacts of organizational stressors on the mental health of police officers, but more specifically the study of moral injuries resulting from police non-operational experiences. For example, what is the impact of an officer risking their life in service of their community while not being supported and valued by their leadership? Although research has advanced beyond simply studying an officer’s work, I believe there is benefit to further the discourse by focusing on an officer’s organizational environment, including leadership. Understanding how moral injuries can result from a perceived incongruence of personal values and leadership actions allows policing advocates to push for improved organizational cultures. The archaic, strongly held tenets of police culture may be influenced by further acknowledgment of how the culture currently breeds leaders who lead operations, not people.

## Conclusion

Organizational stressors are becoming more widely known as having tremendous impact on the psychological wellbeing of police officers (Schafer, 2010; McCreary et al., 2017; Wolter et al., 2019; Carleton et al., 2020; Chan and Andersen, 2020; Demou et al., 2020). Exposure to the trauma of some operational stressors can leave officers less resilient to effectively recognize and address the organizational stressor of ineffective leadership. Further, not only are officers less resilient, but their risk of psychological injuries also increases under this type of organizational stress. Ineffective police leadership is characterized by inconsistency, moral ambiguity, lack of support, oppression, and self-serving agendas, as examples. The pervasiveness of ineffective leadership is further experienced in the paramilitary context of police culture where orders and rank are expected to be followed. Communities require police to be well to effectively address public safety. It is critically important that police leadership be aware of what impacts their officers’ mental health while creating and supporting psychologically safe workplaces.

## Data Availability Statement

Publicly available datasets were analyzed in this study. This data can be found at: https://doi.org/10.1037/1072-5245.13.4.494.

## Author Contributions

This manuscript is an edited original version researched and prepared as part of the author’s masters in counselling psychology requirement for program completion. The original manuscript was written under the supervision of HS of the Graduate Centre for Applied Psychology, Athabasca University.

## Conflict of Interest

The authors declare that the research was conducted in the absence of any commercial or financial relationships that could be construed as a potential conflict of interest.

## Publisher’s Note

All claims expressed in this article are solely those of the authors and do not necessarily represent those of their affiliated organizations, or those of the publisher, the editors and the reviewers. Any product that may be evaluated in this article, or claim that may be made by its manufacturer, is not guaranteed or endorsed by the publisher.
